# 4.2: Vacuum biopsy in the management of lobular *in situ *neoplasia: a single-centre experience

**DOI:** 10.1186/bcr3499

**Published:** 2013-11-08

**Authors:** C Parkin, S Garewal, P Waugh, AJ Maxwell

**Affiliations:** 1 North Western Deanery School of Radiology, Manchester, UK; 2 Royal Bolton Hospital, Bolton, UK; 3 University Hospital of South Manchester, Manchester, UK

## Introduction

Lobular *in situ *neoplasia (LISN) is encountered with increasing frequency in core needle biopsies (CNB) of the breast. It is a generalised risk factor and probable nonobligate precursor for some breast cancers. Historically, open biopsy was performed to exclude associated malignancy. Controversy currently surrounds the management of LISN, and practice consequently varies between departments. This study is a review of a single centre's 13-year experience of managing LISN with vacuum-assisted biopsy (VAB) in order to assess the safety of this policy.

## Methods

A retrospective review of the breast screening database, pathology database, high-risk patient database and patients recruited to the Sloane Project was completed. Patients with LISN as the most pertinent diagnosis on VAB, with or without preceding 14-gauge CNB, were identified. Those with pathological results not concordant with imaging were excluded. The outcome of subsequent annual surveillance mammograms was recorded.

## Results

Between February 1998 and March 2012, 42 patients had LISN as the most pertinent diagnosis at VAB, with or without preceding CNB. No open biopsies were performed in this group. Mean radiological follow up was 39 months (range 0 to 105 months). There were no new diagnoses of breast cancer during follow up. Three patients died: one with a previous history of invasive breast cancer died from metastatic breast cancer and two died from unrelated causes.

## Conclusion

In the presence of adequate tissue sampling and radiological-pathological concordance, VAB is a safe alternative to open biopsy in the management of LISN.

